# Clinical analysis of HIV/AIDS patients with drug eruption in Yunnan, China

**DOI:** 10.1038/srep35938

**Published:** 2016-10-31

**Authors:** Yu-Ye Li, Yong-Mei Jin, Li-Ping He, Jin-Song Bai, Jun Liu, Min Yu, Jian-Hua Chen, Jing Wen, Yi-Qun Kuang

**Affiliations:** 1 Department of Dermatology and Venerology, The First Affiliated Hospital of Kunming Medical University, Kunming 650032, P. R. China; 2 Department of HIV/AIDS, The Third People’s Hospital of Kunming, Kunming 650041, P. R. China; 3 School of Public Health, Kunming Medical University, Kunming 650500, P. R. China; 4 Center for Translational Medicine, Huaihe Clinical College, Henan University, Kaifeng 475000, P. R. China; 5 Pharmaceutical College, Henan University, Kaifeng 475001, P. R. China

## Abstract

Drug eruption is the most common clinical presentation in patients with HIV/AIDS. The systemic clinical and risk factors associated with drug eruption remain unknown. A retrospective analysis in HIV/AIDS patients with drug eruption was carried out with demographic data, epidemiological data, clinical characteristics, laboratory data and follow-up data. The risk factors correlated with prognosis were assessed by case control analysis. A total of 134 out of 1817 HIV/AIDS patients (7.4%) presented drug eruptions. The major class of sensitizing drug was HAART drugs (47.7%), followed by antibiotics (47.0%). Nevirapine (39.6%) was the most common sensitizing drug in the HAART regimens. The patients received HAART or had allergic history were prone to develop drug eruption. The alanine aminotransferase, albumin, globulin, creatinine, blood urea nitrogen (BUN), lymphocytes, red blood cells (RBC) and eosinophils of the drug eruption patients were significantly different the control patients. The allergic history, opportunistic infection, viral load, CD4 cell count, high globulin and low albumin were the risk factors correlated with death in HIV/AIDS patients with drug eruption. It is proposed that patients with higher viral loads, higher globulin levels and lower white blood cells (WBC) should be given special attention for the prevention of complications and death.

In China, the initial HIV-1 outbreak was caused by injection drug use (IDU) in Yunnan in the late 1980s^1^, and Yunnan became the most affected province in China. The key population at high risk of acquiring HIV has progressed from drug users and female sex workers to the general public. HIV has become a serious threat to public health and an important limiting factor for social stability and economic development^2^. In 1987, the first antiretroviral drug, Zidovudine (AZT), was available to AIDS patients in clinics. Currently, a stable and effective combination therapy, highly active antiretroviral therapy (HAART), has been developed and has fundamentally changed the number of individuals progressing to AIDS and greatly reduced the morbidity and mortality of HIV/AIDS^3^. In China, a free HAART treatment policy was implemented by the government beginning in 2003. Currently, these drugs are extensively used nationwide. The first-line HAART treatment regimen is AZT/D4T/TDF + 3TC + NVP/EFV, and the second-line treatment regimen is AZT/TDF + 3TC + LPV/r^4^.

With the increasing application of HAART throughout the world, the quality of AIDS patients’ lives has been improved, the survival time is prolonged, and the lives of numerous AIDS patients have been saved. However, HAART regimens cannot purge the HIV-1 virus in patients and completely cure AIDS, and side effects occur with clinical therapies, which leads to the patient’s termination of treatment and even death^5^. Although HAART reduces the incidence of mucocutaneous disorders in HIV-1-infected patients^6^,^7^, the treatment regimen itself could be the cause of a common side effect, the drug eruption^8^. AIDS patients may also experience various opportunistic infections and tumors, and thus they also take additional drugs, including several types of antibiotics, antifungal drugs, anti-infective drugs, and even antitumor drugs, which increases the risk of drug eruption^9^,^10^. In these cases, for AIDS patients, a systematic study of drug eruption, including types of drug eruption and drug eruption-related factors influencing prognosis, is of crucial clinical significance.

In this study, we conducted a retrospective analysis of drug eruptions among HIV/AIDS patients in Yunnan Province, China. We explored the drugs causing allergies in HIV/AIDS patients in this region combined with drug eruptions and analyzed the types of drug eruptions as well as the risk factors affecting prognosis. The data from this study provide a theoretical basis and practical guide for the prevention and treatment of HIV/AIDS patients with drug eruption.

## Results

### Demographic Data of Participants

A total of 1817 HIV/AIDS patients were hospitalized at the Third People’s Hospital of Kunming City from January 2011 to December 2013. Among them, 134 patients were hospitalized because of drug eruption or presented drug eruption during their hospitalization. The incidence of drug eruption in HIV/AIDS patients was 7.4%. In the patients with drug eruption, 54.5% (73/134) of HIV infections were found because of AIDS symptoms, and 45.5% (61/134) of infections were identified by physical examination. A 1:2 random sample (268 cases) of hospitalized AIDS/HIV patients without drug eruption was used as the control group (Table 1). Detailed demographic information of the drug eruption group was recorded (Table S1). No significant difference was observed in the age and sex distribution between the two groups (Table 1).

### Immunological, virologic, and antiviral treatment status of the participants

#### CD4^+^ T cell count

The host immunological status was evaluated by CD4^+^ T cell count. In the drug eruption group, the median ± interquartile range (IR) of the CD4^+^ T cell count was 151.00 ± 221.50. In the control group, the median ± IR of the CD4^+^ T cell count was 135 ± 246.75. There was no significant difference observed in the CD4^+^ T cell count between the drug eruption group and the control group (*P* = 0.267) (Table 1).

#### HIV-1 viral load

The virological status was evaluated by viral load quantitation. In the drug eruption group, the median ± IR of the HIV-1 viral load was 3220.00 ± 361,450.00. In contrast, the median ± IR of the HIV-1 viral load was 27,650.00 ± 461,950.00 in the control group. The HIV viral load in the drug eruption group was not significantly higher than the control group (*P* = 0.436) (Table 1).

#### HAART treatment

A total of 61.2% (82/134) of patients with drug eruption were treated with HAART, and 71.6% (192/268) of patients in the control group were treated with HAART. The proportion receiving HAART treatment in the drug eruption group was significantly lower than that in the control group (*P* = 0.034) (Table 1).

#### Drug allergy history

A total of 15.7% (21/134) of patients in the drug eruption group had a drug allergy history, which was significantly higher than the control group (20/268, 7.5%, *P* = 0.010) (Table 1).

### Clinical characteristics and laboratory testing data

The latency and the causative sensitizing drugs were analyzed among the drug eruption patients (Table S2). The average latency period for the drug eruption was 10.78 ± 7.28 days. A total of 47.7% (64/134) of patients in the drug eruption group were sensitized by the HAART drugs. The non-nucleoside reverse transcriptase inhibitor (NNRTI), Nevirapine, was the most common drug involved in sensitization (39.6%, 53/134), followed by Efavirdine (9.0%, 12/134) (Table S2).

#### The status of opportunistic infections

A total of 61.2% (82/134) of patients with drug eruption were affected by opportunistic infections. Approximately 78.4% (105/134) of HIV/AIDS patients were co-infected by bacteria, followed by fungal infections (53.0%, 71/134), viral infections (20.1%, 28/134) and parasitic infections (1.5%, 2/134). Bacterial pneumonia and tuberculosis were the most common bacterial infections. Candidiasis was the most common fungal infection agent, followed by pneumocystis pneumonia. A total of 16 cases (11.9%) and 4 cases (3%) were co-infected by HCV and HBV, respectively (Table S3).

#### Systemic symptoms and damage among drug eruption patients

Hepatic dysfunction occurred in 70.1% (94/134) of patients in the drug eruption group, which is significantly higher than in the control group (*P* = 0.014) (Table 2). Liver metabolism disorders occurred mainly as toxic hepatitis, intrahepatic cholestasis, or elevated levels of liver enzymes such as total bilirubin (t-Bil), aspartate aminotransferase (AST), alanine transaminase (ALT), ALB, and globulin (GLB). Among the drug eruption group, the median ± IR of the ALT, ALB and GLB levels in the serum were 41.00 ± 86.50, 35.85 ± 7.05 and 29.85 ± 8.18, respectively, which was significantly higher than those in the control group (*P* < 0.001) (Table 2).

Renal dysfunction occurred in 9% (12/134) of patients in the drug eruption group. The renal function tests included serum creatinine (CREA) and urea nitrogen (BUN). Among them, the median ± IR serum CREA and BUN levels were 63.00 ± 21.50 and 3.60 ± 2.49, which was significantly lower than the control group (Table 2).

The routine examination of the blood to evaluate damage to the hematologic system showed that in the drug eruption group, the median ± IR of lymphocyte (LYM), red blood cell (RBC) and eosinophil (EOS) numbers were 0.98 ± 0.81, 3.95 ± 0.69 and 0.10 ± 0.22, respectively, which was significantly higher than the control group (*P* < 0.05) (Table 2).

### The prognosis of patients with drug eruption

A total of 10 cases (7.5%) out of 134 HIV/AIDS patients with drug eruption died, including 6 out of 29 patients (20.6%) with severe drug eruption and 4 out of 105 patients (3.8%) with mild drug eruption. Among the patients with drug eruption, the risk factors correlated to the prognosis of the patients were analyzed. The mortality of patients with allergic history (*P* = 0.008) and opportunistic infections was significantly higher (*P* = 0.009). In addition, the median ± IR viral load (1950.00 ± 288,700.00) or CD4^+^ T cell count (157.50 ± 219.25) of the cured/improved case group was significantly lower than the death case group (*P* = 0.008 and *P* = 0.021, respectively) (Table 3).

Next, the effects of the hepatic metabolism indexes, renal function and the hematologic system on the prognosis were evaluated. The median ± IR ALB (36.15 ± 6.35), RBC (4.00 ± 0.67) and Hb (121.54 ± 18.25) values in the cured/improved case group were significantly higher than those in the death case group (*P* = 0.002, *P* = 0.002 and *P* < 0001, respectively). There was no significant difference in other patient factors between the compared groups (Table 3).

Lastly, the variables of the patients in the drug eruption group were analyzed using logistic regression analysis. The results showed that the prognostic index was affected by the viral load ≥1 × 10^2^ copies/mL, high GLB and low WBC. As could be observed from the OR value, after eliminating the other confounding factors, for one order of magnitude increase of the viral load, and the GLB and WBC values in HIV/AIDS patients with drug eruption, the patients were 3.237-fold, 0.114-fold and 0.068-fold more likely to die than the cured or improved patients, respectively (Table 4).

## Discussion

Adverse drug reactions, including drug eruption, have been observed in HIV/AIDS patients^11^,^12^,^13^,^14^. The negative effects caused by drug eruption result in the early discontinuation of therapy in patients. However, the risk factors of drug eruptions in response to drugs (not only HAART drugs) in AIDS patients are not well understood^12^. Understanding the mechanisms of drug eruption and improving the therapeutic strategies are of great importance in clinic. In this study, drug eruption was observed in every stage of the HIV/AIDS course in patients. In the drug eruption group, 60.4% of patients had a CD4^+^ T cell count ≤ 200 cells/μL, 70.9% of patients had a detectable viral load, and 61.2% of patients were enrolled in HAART regimens. Approximately 61.2% of HIV/AIDS patients with drug eruption were affected by opportunistic infections. Most HIV/AIDS patients were affected by opportunistic infections and were often treated for these infections^15^,^16^.

The rate of drug allergic history in the drug eruption group was significantly higher than in the control group (*P* = 0.010, Table 1). This suggests that patients with a history of drug hypersensitivity are more likely to have drug eruptions. Before treating patients with new medications, doctors should inquire about the patient’s drug hypersensitivity history and avoid using medicines that may cause allergies or structural analogs of those medicines. Approximately 47.7% of patients with drug eruption were sensitized by HAART drugs, followed by antibacterial drugs (45.5%). In patients with HIV infection, antibiotics are the leading drugs that cause allergies^17^,^18^. Among HAART regimens, 46.2% of patients were sensitized by NNRTIs, of which 26.6% of patients progressed to severe drug eruption. Nevirapine was the most common drug involved in sensitization (39.6%), followed by Efavirdine (9.0%). These data suggest that Nevirapine is the major drug causing allergy, in which mild drug eruption at an early stage might develop into severe drug eruption^19^,^20^.

Close monitoring for drug eruption is required for HIV/AIDS patients treated with HAART, especially for the first month in patients taking Nevirapine^21^, in order to ensure that those patients stop taking the medication or treatment in time to prevent the patients from progressing from mild drug eruption to severe drug eruption. The present studies recommend the use of Efavirdine instead of Nevirapine^22^. Efavirdine and Nevirapine are both NNRTI analogues with similar structures. The HLA-DRB1*01 allele was reported to be associated with the susceptibility to cutaneous reactions associated with these two drugs in HIV-1-infected French patients^23^. When Nevirapine is changed to Efavirdine, monitoring for cross-allergic reactions should be strengthened. Interestingly, we also found that one patient was allergic to Tenofovir ester, which was rarely reported previously. Because of the safety and the anti-HBV effect of Tenofovir ester^24^, the Chinese government provides it as a free first-line antiviral drug^4^. Lockhart *et al*. also reported 9 cases of HIV-positive patients with drug eruption after treatment with Tenofovir ester^25^, and thus patients should also be monitored for drug eruption with this drug. Notably, one case in the drug eruption group was allergic to Lopinavir/Ritonavir, which belongs to the PI combination group; its sensitization has not yet been reported in China.

A total of 45.5% of patients in the drug eruption group were allergic to antibacterial drugs. This is pertinent for the antimicrobial treatment for 78.4% of patients with bacterial co-infections^26^, and it also affects the adherence to antibiotic drugs in China^18^. Furthermore, 9.7% of patients were allergic to anti-tuberculosis drugs, of which the most common sensitizing drug was Rifamycin. A total of 35.8% of patients in the drug eruption group were co-infected by mycobacteria, of which 34.5% were infected by tuberculosis. Thus, this allergy affects patients with a common opportunistic mycobacteria co-infection and using anti-tuberculosis medicine^27^. Rifamycin is known as a necessary component of an anti-tuberculosis treatment program, and thus the occurrence of drug eruption should be under intensive monitoring over the first three weeks. The second most common sensitizing anti-tuberculosis drug was the quinolone class (8.2%), of which most patients received levofloxacin. Levofloxacin is a broad-spectrum antibiotic, and HIV/AIDS patients with tuberculosis are typically treated with Levofloxacin as a second-line anti-tuberculosis drug^28^,^29^. A total of 5.2% of patients were sensitized by sulfonamides, which is also one of the most common types of clinical sensitizing drugs^30^. A total of 15.7% of patients have pneumocystis jiroveci pneumonia and 1.5% have toxoplasmosis encephalitis, and sulfonamides are the first-choice treatment for them^31^. The WHO and UNAIDS recommend it as a preventive medicine for people living with HIV and AIDS worldwide.

In addition to drug eruption, most patients presented systemic damage, including fever (35.1%), acute drug-induced liver damage (65.7%), renal dysfunction (9.0%) and hematopoietic damage. Among these, acute drug-induced liver damage was identified as an increase in t-Bil, AST and ALT in 20.9%, 57.5% and 53.7% of patients, respectively. Close monitoring for liver clinical manifestations such as anorexia, intolerance of fatty foods, nausea, vomiting and jaundice is recommended. There was a decrease in the WBC, NEU, LYM, RBC, Hb and PLT values in 52.2%, 35.2%, 34.3%, 56.0%, 41.0% and 11.0% of patients, respectively, while EOS was increased in 11.2% of patients. Drug eruption can cause anemia and a decrease in WBC and PLT but an increase in EOS. The incidence of fever and hepatic dysfunction in HIV/AIDS patients is higher than in non-HIV/AIDS patients^32^. The data suggest that systemic damage should be evaluated when HIV/AIDS patients experience drug eruption, and the status of their liver function and kidney function and routine blood tests should be strictly monitored. A total of 41.8% of patients had hypoalbuminemia (data not shown), which indicates that supportive treatment should be performed in a timely fashion.

Approximately 29.9% of patients’ drug eruption reactions were cured after general treatment. The mortality among patients with severe drug eruption was 20.7%, which is higher than the reported mortality of non-AIDS patients (9.68%). The mortality of patients with severe drug eruption, drug hypersensitive history, CD4^+^ T cell count ≤200 cells/μL, opportunistic infection, viral load ≥1 × 10^2^ copies/mL, low ALB level or decreased RBC and Hb was significantly increased. Severe drug eruption, high viral load, high GLB level and elevated WBC count could be considered as the risk factors leading to death. Severe drug eruption is an important factor causing death in HIV/AIDS patients, and these patients require active and comprehensive treatment.

Taken together, the incidence of drug eruption was 7.4% among the treated HIV/AIDS patients. HAART drugs were the major drugs causing allergies in HIV/AIDS patients. The fatality rate among HIV/AIDS patients with drug eruption was higher than in the patients without drug eruption, especially for the patients with severe drug eruption. Our data imply that drug eruption patients with viral load ≥10^2^ cpm, high globulin levels and low WBC count should be actively treated as soon as possible. This study provides a theoretical basis for the prevention and treatment of HIV/AIDS patients with drug eruptions.

## Materials and Methods

### Ethics Statement

This study was approved by the First Affiliated Hospital of Kunming Medical University, and written informed consent was obtained from the study participants. All experiments were performed in accordance with the approved guidelines and regulations, and the experimental protocols were approved by the institutional review boards of Kunming Medical University and Henan University.

### Subjects

Subjects were recruited at the AIDS division of the Third People’s Hospital of Kunming City. The drug eruption group (n = 134) was collected from 1817 AIDS patients who were hospitalized for drug eruptions or developed drug eruptions during hospitalization. A group of AIDS patients without drug eruptions was used as a control group (n = 268). The demographic information, epidemiological data, clinical features, laboratory examination data and disease prognosis were obtained. Drug eruption was classified and treated accordingly. One month later, follow-up was conducted to determine whether the patient lived or died.

### CD4^+^ T cell counting

CD4^+^ T lymphocyte counts were measured by a FACSCalibur^TM^ flow cytometer (BD Bioscience, CA, USA) using a Multitest^TM^ IMK Kit (BD Bioscience, CA, USA). The CD4^+^ T lymphocyte count was measured in cells/μL.

### Viral load

HIV-1 RNA was quantified using a DA7600 Real Time PCR machine and its supporting HIV-1 PCR Kit (DAAN gene Co., China). The viral load was measured in copies/mL, and the limit of detection was 1 × 10^2^ copies/mL (cpm).

### Viral hepatitis serological test

HAV-Ab, HBV-M, HCV-Ab, HDV-Ab, and HEV-Ab were determined by enzyme-linked immunosorbent assay (Huakang).

### Test of hepatic and renal functions

Hepatic functions, including the total bilirubin (t-Bil), aspartate aminotransferase (AST), alanine transaminase (ALT), albumin (ALB), and globulin (GLB), were tested. The renal function tests included serum creatinine (CREA) and urea nitrogen (BUN) and were measured using an AU5400 automatic biochemical analyzer (Olympus).

### Routine blood test

Hematological analysis, including white blood cells (WBC), neutrophils (NEU), lymphocytes (LYM), red blood cells (RBC), hemoglobin (Hb), platelets (PLT), and eosinophils (EOS), was performed with an XE5000 automated hematology analyzer (Sysmex).

### Diagnosis of drug eruption

The diagnosis and classification of drug eruption was determined by combining laboratory tests with the medical history, the latency period, and the morphology and distribution of the rash. Any diseases with similar symptoms were excluded, such as measles and scarlet fever. According to the characteristics of the rash and laboratory examination, drug eruptions were divided into different types, including severe erythema multiforme type, bullosa epidermolysis necrosis drug eruption, erythrodermic type, and drug hypersensitivity syndrome.

### Statistical analysis

A database was built using Excel and statistically analyzed using SPSS 17.0 software. The normal distribution of the data is represented by the mean ± standard deviation. The comparison of two mean values was analyzed using a *t*-test, and multiple mean values were compared using an analysis of variance. The skewness distribution of the data is represented by the median (M) and interquartile range (*P*25, *P*75) using a rank sum test. The numerical data among groups were analyzed using a χ^2^ test. Fisher’s exact probability was used when the theoretical value was less than 1. Logistic regression was used to analyze the multiple influencing factors. *P* < 0.05 was defined as statistically significant. To calculate the *P* value among multiple groups, the comparison between each pair of groups was conducted, and the significance level was 0.05 divided by the number of tests.

## Additional Information

**How to cite this article**: Li, Y.-Y. *et al*. Clinical analysis of HIV/AIDS patients with drug eruption in Yunnan, China. *Sci. Rep.* **6**, 35938; doi: 10.1038/srep35938 (2016).

**Publisher’s note:** Springer Nature remains neutral with regard to jurisdictional claims in published maps and institutional affiliations.

## Supplementary Material

Supplementary Information

## Figures and Tables

**Table 1:** Demographic data, CD4^+^ T cell count, viral load and HAART treatment of studied subjects.

		Drug eruption group n = 134 (%)	Control group n = 268 (%)	*P* value
Age	Mean ± SD	41.43 ± 12.39	43.31 ± 12.96	0.164
	≤30	27 (20.1)	38 (14.2)	0.460
	31–40	49 (36.6)	89 (33.2)	
	41–50	29 (21.6)	85 (31.7)	
	51–60	17 (12.7)	24 (9.0)	
	>60	12 (9.0)	32 (11.9)	
Sex	Male	89 (66.4)	199 (74.2)	0.100
	Female	45 (33.6)	69 (25.8)	
CD4^+^ T cell count	Median ± IR^b^	151.00 ± 221.50	135.00 ± 246.75	0.267
HIV-1 viral load	Median ± IR	3220.00 ± 361450.00	27650.00 ± 461950.00	0.436
HAART	Yes	82 (61.2)	192 (71.6)	0.034^a^
	No	52 (38.8)	76 (28.4)	
Allergic history	Yes	21 (15.7)	20 (7.5)	0.010^a^
	No	113 (84.3)	248 (92.5)	

^a^*P* < 0.05.

^b^IR = interquartile range.

**Table 2:** The distribution of clinical indexes between the drug eruption and control groups.

		Drug eruption group n = 134	Control n = 268	*P* value
Hepatic dysfunction	Yes	94 (70.1)	114 (42.5)	0.014^**a**^
	No	40 (29.9)	154 (57.5)	
t-Bil	Median ± IR	8.95 ± 8.75	10.45 ± 8.95	0.467
AST	Median ± IR	45.00 ± 66.25	41.00 ± 50.75	0.212
ALT	Median ± IR	41.00 ± 86.50	25.00 ± 36.00	<0.001^**a**^
ALB	Median ± IR	35.85 ± 7.05	32.25 ± 12.45	0.002^**a**^
GLB	Median ± IR	29.85 ± 8.18	33.25 ± 11.90	<0.001^**a**^
Kidney dysfunction	Yes	12 (9.0)	41 (15.3)	0.076
	No	122 (91.0)	227 (84.7)	
CREA	Median ± IR	63.00 ± 21.50	70.00 ± 29.00	0.002^**a**^
BUN	Median ± IR	3.60 ± 2.49	4.10 ± 2.76	0.006^**a**^
WBC	Median ± IR	3.90 ± 2.47	3.91 ± 3.05	0.634
NEU	Median ± IR	2.28 ± 2.02	2.45 ± 2.37	0.189
LYM	Median ± IR	0.98 ± 0.81	0.86 ± 0.86	0.039^**a**^
RBC	Mean ± SD	3.95 ± 0.69	3.70 ± 0.96	0.003^**a**^
Hb	Median ± IR	119.50 ± 24.25	117.00 ± 39.75	0.233
PLT	Median ± IR	163.00 ± 104.00	143.50 ± 108.75	0.090
EOS	Median ± IR	0.10 ± 0.22	0.05 ± 0.11	0.001^**a**^

Abbreviations: t-Bil, total bilirubin; AST, aspartate aminotransferase; ALT, alanine aminotransferase; ALB, albumin; GLB, globulin; CREA, creatinine; BUN, blood urea nitrogen; WBC, white blood cells; NEU, neutrophils; LYM, lymphocytes; RBC, red blood cells; Hb, hemoglobin; PLT, platelets; EOS, eosinophils.

^a^*P* < 0.05.

^b^IR = interquartile range.

**Table 3:** Correlation analysis of risk factors with the prognosis of patients with drug eruption.

Risk factor		Drug eruption group (n = 134)		*P* value
		Cured/improved cases (n = 124)	Death cases (n = 10)	
Sex	Male	83 (93.3)	6 (6.7)	0.679
	Female	41 (91.1)	4 (8.9)	
Age	≤50	98 (92.5)	8 (7.5)	0.942
	>50	26 (92.9)	2 (7.1)	
Allergic history	Yes	16 (76.2)	5 (23.8)	0.008^a^
	No	108 (95.6)	5 (4.4)	
Opportunistic infection	Yes	72 (77.8)	10 (12.2)	0.009^a^
	No	52 (100.0)	0 (0.0)	
HAART	Yes	78 (95.1)	4 (4.9)	0.153
	No	46 (88.5)	6 (11.5)	
Sensitization route	Intravenous	38 (88.4)	5 (11.6)	0.363
	Oral	86 (94.5)	5 (5.5)	
Fever	Yes	43 (93.0)	4 (7.0)	1.000
	No	81 (92.2)	6 (7.8)	
Lymphadenectasis	Yes	7 (87.5)	1 (12.5)	0.472^b^
	No	117 (92.9)	9 (7.1)	
Viral load	Median ± IR^c^	1950.00 ± 288700.00	440000.00 ± 1917225.00	0.008^a^
CD4^+^ T cell count	Median ± IR	157.50 ± 219.25	41.50 ± 122.25	0.021^a^
t-Bil	Median ± IR	8.70 ± 8.70	12.30 ± 10.40	0.400
AST	Median ± IR	45.00 ± 69.25	44.00 ± 32.00	1.000
ALT	Median ± IR	42.50 ± 87.00	39.00 ± 86.25	0.684
ALB	Median ± IR	36.15 ± 6.35	31.35 ± 13.00	0.002^a^
GLB	Median ± IR	29.95 ± 8.33	29.05 ± 8.60	0.694
CREA	Median ± IR	63.00 ± 20.00	59.80 ± 40.50	0.531
BUN	Median ± IR	3.60 ± 2.25	5.75 ± 4.37	0.192
WBC	Median ± IR	3.97 ± 2.53	3.15 ± 1.83	0.041^a^
NEU	Median ± IR	2.42 ± 2.10	1.95 ± 1.49	0.207
LYM	Median ± IR	1.00 ± 0.85	0.80 ± 0.47	0.109
RBC	Mean ± SD	4.00 ± 0.67	3.31 ± 0.60	0.002^a^
Hb	Mean ± SD	121.54 ± 18.25	98.10 ± 18.53	<0.001^a^
PLT	Mean ± SD	166.62 ± 74.60	148.00 ± 72.21	0.448
EOS	Median ± IR	0.10 ± 0.22	0.11 ± 0.45	0.760

^a^*P* < 0.05.

^b^Fisher’s exact test.

^c^IR = interquartile range.

**Table 4:** Logistic regression analysis of death factors.

	B	S.E.	Wald	df	*P* value	OR	95% C.I. for Exp (B)	
							Lower	Upper
Viral load ≥10^2^ cpm	1.175	0.392	8.987	1	0.003^a^	3.237	1.502	6.977
High GLB	−2.173	1.079	4.053	1	0.044^a^	0.114	0.014	0.944
Low WBC	−2.682	1.115	5.788	1	0.016^a^	0.068	0.008	0.608
Constant	−10.118	3.959	6.532	1	0.011^a^	0.000		

^a^*P* < 0.05.
