# Supplementary material for: Clinical analysis of HIV/AIDS patients with drug eruption in Yunnan, China
Source: Sci Rep. 2016 Oct 31;6:35938. doi: 10.1038/srep35938 (PMC5086857; doi:10.1038/srep35938)
Supplement: Supplementary Information [file srep35938-s1.pdf]

Clinical analysis of HIV/AIDS patients with drug eruption in Yunnan, China

Yu-Ye Li[1,*], Yong-Mei Jin[1,2,*] Li-Ping He[3], Jin-Song Bai[2], Jun Liu[2], Min Yu[2], Jian-Hua Chen[2], Jing Wen[4,5] & Yi-Qun Kuang[4]

1. Department of Dermatology and Venerology, The First Affiliated Hospital of Kunming Medical University, Kunming 650032, P. R. China
2. Department of HIV/AIDS, The Third People's Hospital of Kunming, Kunming 650041, P. R. China
3. School of Public Health, Kunming Medical University, Kunming 650500, P. R. China
4. Center for Translational Medicine, Huaihe Clinical College, Henan University. Kaifeng 475000, P. R. China
5. Pharmaceutical College, Henan University, Kaifeng 475001, P. R. China

*These authors contributed equally to this work.

Correspondence should be addressed to Y.Q.K. (E-mail: yqkuang@henu.edu.cn) or Y.Y.L. (E-mail: yyeli2000@126.com).

Supplemental Table S1. Demographic data of patients with drug eruptions

		Drug eruption (n = 134)	(%)
Residence	Local	119	88.8
	Out of the province	15	11.4
Nationality	Han	126	92.1
	Yi	2	2.0
	Hui	2	2.0
	Bai	2	4.0
	Uyghur	1	0.7
	Zhuang	1	0.7
Marriage	Married	96	71.6
	Unmarried	28	20.9
	Divorced	6	4.5
	Widowed	4	3.0
Occupation	No occupation	40	29.9
	farmer	36	26.9
	worker	22	16.4
	Company worker	12	9.0
	Small businessman	9	6.7
	Government officer	7	5.2
	Migration worker	5	3.7
	student	2	1.5
	Driver	1	0.7
Education	Elementary high school	24	17.9
	Junior high School	57	42.5
	Senior school/	30	22.4
	College	23	17.2
Transmission route	Heterosexual	89	66.4
	IDU	21	15.7
	Homosexual	16	11.9
	Unknown	8	6.0
Spouse infection	No	75	56.0
	No spouse	33	24.6
	Yes	26	19.4
Opportunistic infection	Yes	83	61.9
	No	51	38.1
Viral hepatitis	HBV	16	11.9
	HCV	4	3.0

Supplemental Table S2. List of sensitizing drugs causing the eruption in patients

Sensitizing drug			Case number (%)	Latency (day)	Drug list (case number)
HAART	NNRTI		62 (46.2)	13.91 ± 5.99	NVP (50), EFV (9), NVP+EFV (3)
	NNNRTI		2 (1.5)		LPV/Ritonavir (1), TDF (1)
Antimicrobial	B-Lactams	Cephalosporins	23 (17.2)	6.13 ± 4.51	Cefpirome (7), Cefathiamidine (5), Cefodizime (3), Ceftriaxone (2), Cefepime (1), Sulbactam and Cefopcrazone (1), other Cephalosporins (4)
		Penicillins	1 (0.7)	3	Ampicillin (1)
		Carbapenems	1 (0.7)	12	Meropenem (1)
	Antituberculotic		13 (9.7)	14.15 ± 7.24	Rifampicin (5), Rifabutin (4), Isoniazid (3), Rifabutin+Pyrazinamide (1)
	Quinolone		11 (8.2)	4.18 ± 2.04	Levofloxacin (9), Moxifloxacin (2)
	Sulfanmide		7 (5.9)	8.38 ± 10.66	Sulfamethoxazole (6), Belladonna Sulfamethoxazole and Trimerhoprim/Imodium (1)
	Macrolide		2 (1.5)	14.0 ± 13.9	Azithromycin (1), Clarithromycin (1)
	Polypeptide		1 (0.7)	2	Vancomycin (1)
	Lincosamide		1 (0.7)	14	Clindamycin (1)
Other antiviral drugs			1 (0.7)	6	Ganciclovir (1)
Anti-fungi			1 (0.7)	1	Itraconazole (1)
Chinese medicine			3 (2.2)	7.0 ± 5.2	Decotion (2), Qingkailing (1)
Antigout			2 (1.5)	17.0 ± 16.9	Allopurinol (1), Benzbromarone (1)
Bioproduct			1 (0.7)	3	Human G-CSF (1)
nutriceutical			1 (0.7)	2	Compound Amino Acid Injection/18AA (1)
			1 (0.7)	3	Tiopronin (1)

Abbreviationss: NVP, nevirapine; EFV, efavirenz; LPV, lopinavir; TDF, tenofovir disoproxil fumarate.

Supplemental Table S3. Opportunistic infections by pathogens among drug eruption group

Pathogen	Disease	Number (%)
Bacterium	bacterial pneumonia	50 (37.3)
	Tuberculosis	46 (34.3)
	bacterial septicemia	5 (3.7)
	cellulitis	2 (1.5)
	mycobacterium tuberculosis	2 (1.5)
Fungi	Candidiasis	41 (30.6)
	pneumocystis pneumonia	21 (15.7)
	Penicilliosis marneffei	7 (5.2)
	Cryptococcosis	2 (1.5)
Virus	HBV	16 (11.9)
	HCV	4 (3.0)
	HSV	4 (3.0)
	CMV	2 (1.5)
	VZV	2 (1.5)
Parasite	toxoplasmosis encephalitis	2 (1.5)

Abbreviations: HBV, hepatitis B virus; HCV, hepatitis C virus; HSV, herpes simplex virus; CMV, cytomegalovirus; VZV, varicella zoster virus.
